# An Anaplastic Pleomorphic Variant of Mantle Cell Lymphoma

**DOI:** 10.1002/jha2.70084

**Published:** 2025-06-17

**Authors:** Radu Chiriac, Marie Donzel

**Affiliations:** ^1^ Hematology Laboratory, Hospices Civils de Lyon, Centre Hospitalier Lyon Sud Lyon France; ^2^ Pathology Department Hospices Civils de Lyon, Centre Hospitalier Lyon Sud Lyon France

**Keywords:** mantle cell lymphoma, pleomorphic, t(11;14), TP53

1

A 54‐year‐old man presented with a 3‐week history of fatigue and progressively enlarging cervical lymph nodes. Initial blood tests revealed anemia (68 g/L), leukocytosis (25 × 10⁹/L), and a peripheral blood smear showing features of leukoerythroblastosis, with rare (10%) circulating blast‐appearing cells characterized by open chromatin and nuclear irregularities (Figure 1A). Elevated lactate dehydrogenase (LDH) levels (940 U/L) were also noted. Imaging revealed extensive lymphadenopathy above and below the diaphragm, along with hepatosplenomegaly.

**FIGURE 1:**
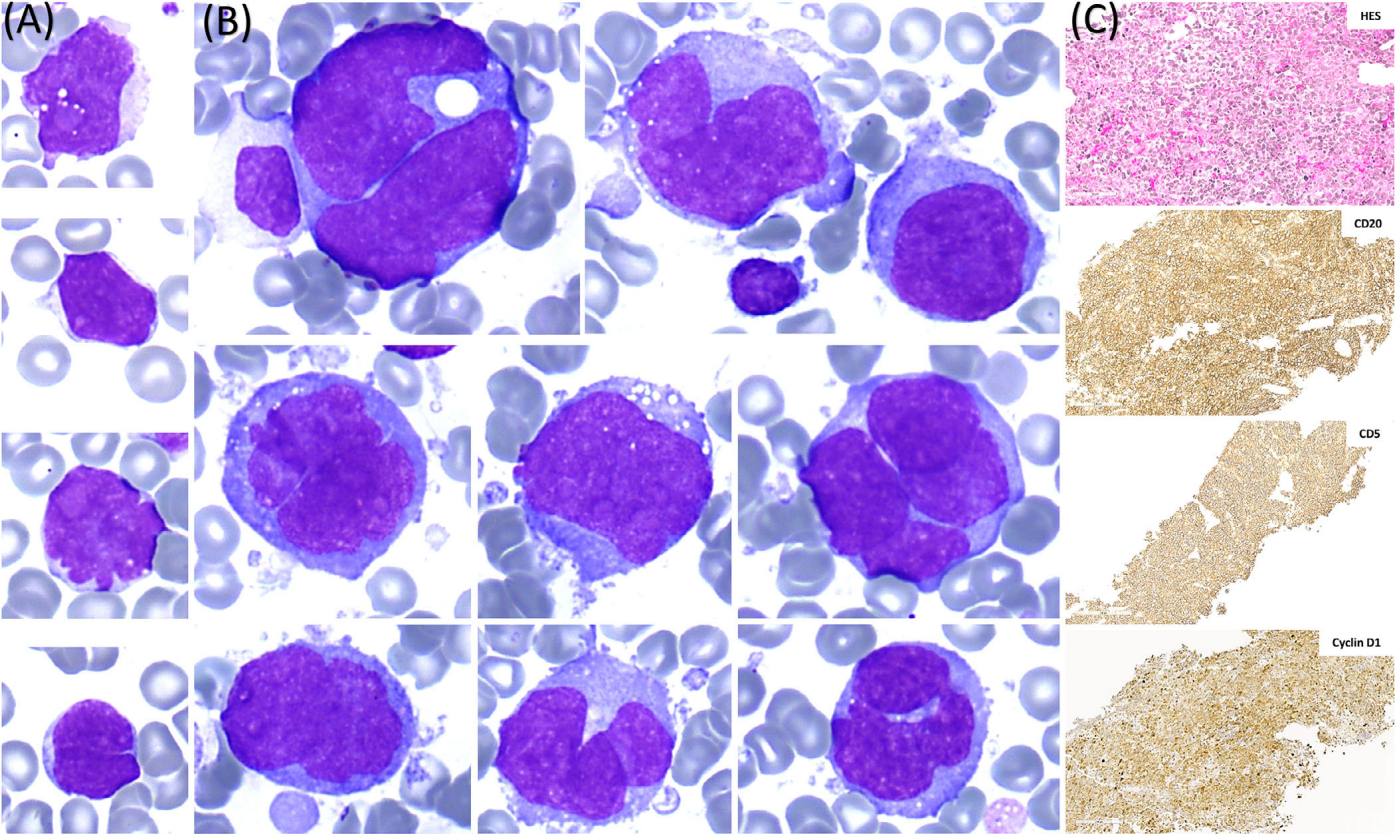
(A) MGG stain, ×100 objective, peripheral blood smear showing rare atypical lymphoid cells with nuclear irregularities. (B) MGG stain, ×100 objective, bone marrow aspirate showing large pleomorphic lymphoid cells of varying size, with nuclear irregularity, and basophilic, vacuolated cytoplasm. (C) Lymph node biopsy, ×10 objective, revealing diffuse lymphomatous proliferation composed of medium to large‐sized cells that were positive for CD5, CD20, and CCND1.

Bone marrow aspirate revealed a massive infiltrate of large pleomorphic and highly atypical lymphoid cells of varying size, with nuclear irregularity, open chromatin, prominent nucleoli, and basophilic, vacuolated cytoplasm (Figure 1B). Flow cytometry identified an increased forward and side scatter monoclonal kappa B‐cell population (CD5+, CD10‐, CD20+, CD23‐, and CD200‐).

A cervical lymph node biopsy (Figure 1C) confirmed the pleomorphic variant of mantle cell lymphoma (MCL), revealing diffuse lymphomatous proliferation of predominantly large cells with mild anaplasia. No areas of pronounced anaplasia were observed. The cells were positive for CD20, PAX5, CD19, CD5, BCL2, CCND1, and SOX11, and negative for CD10 and BCL6. EBER in situ hybridization was negative. The Ki‐67 proliferation index was 80%, with P53 overexpression and no c‐MYC overexpression. Targeted next‐generation sequencing identified a *TP53* c.535C>G (p.His179Asp) mutation with a variant allele frequency of 83%. Both bone marrow and lymph node karyotyping revealed a hypertriploid karyotype (70–73 chromosomes) with t(11;14)(q13;q32) and significant abnormalities, including multiple chromosomal losses (Y, 9, 11, 12, 13, 14, 15, 18, 21, 22), gains (4, 16, 19, 20), structural additions (1p34, 1q11, 3p11, 7p11, 8p11, 22p11), and deletions (9q11, 17p12).

High‐risk features at diagnosis included stage IV A disease, a high‐risk biological MCL International Prognostic Index (MIPI) score of 9, and skeletal involvement. No cerebrospinal fluid infiltration was noted at diagnosis.

The patient relapsed with gastrointestinal involvement one year after receiving four cycles of R‐DHAOx (rituximab, dexamethasone, cytarabine, oxaliplatin), followed by R‐BEAM (rituximab, carmustine, etoposide, cytarabine, melphalan), autologous stem cell transplantation, and maintenance rituximab. He was started on autologous anti‐CD19 chimeric antigen receptor (CAR) T‐cell therapy (brexucabtagene autoleucel), and an 18F‐fluorodeoxyglucose positron emission tomography/computed tomography performed one month after infusion revealed a complete metabolic response. At the time of writing, the patient remains in complete metabolic response at 7 months post‐CAR T‐cell infusion. Continued follow‐up will be necessary to assess the durability of this response and long‐term prognosis.

The distinctive feature of this case is the remarkable morphological findings, which are regarded as highly unusual even within the context of pleomorphic MCL.

Pleomorphic MCL often exhibit complex karyotypic abnormalities. Specific chromosomal abnormalities, such as those involving chromosome 17, are more commonly associated with certain morphological variants, like those with prominent nucleoli. Additionally, tetraploid chromosomal clones are more frequently observed in the pleomorphic variant compared to typical MCL [1]. These abnormalities likely arise later in MCL development, with pleomorphic variants stemming from distinct molecular pathways. The t(11;14) translocation and cyclin D1 overexpression alone are insufficient for MCL, emphasizing the role of additional molecular changes. Pleomorphic MCL shows greater chromosomal instability, including more frequent tetraploid clones [1].

This case highlights multiple high‐risk features in MCL—namely *TP53* mutation, high Ki‐67 index, pleomorphic morphology, complex hypertriploid karyotype, elevated LDH, and a high‐risk MIPI score. Notably, *TP53* mutation and high proliferation are linked to poor response to conventional treatments and early relapse. These findings underscore the need for intensified therapeutic strategies, such as CAR T‐cell therapy, to improve patient outcomes [2].

## Author Contributions

**Radu Chiriac** and **Marie Donzel** wrote the manuscript and conducted the cytomorphological examination. All authors contributed to the final manuscript.

## Conflicts of Interest

The authors declare no conflicts of interest.

## Ethics Statement

This manuscript respects the ethical policy of CHU Lyon for the treatment of human research participants.

## Consent

The authors have confirmed that a patient consent statement is not required for this submission, as no patient‐identifying data were used.

## Clinical Trial Registration

Not applicable.

## Data Availability

Data sharing is not applicable to this article as no new data were created or analyzed in this study.
